# Stroke: Risk Factors, Mechanisms, Outcomes and Ethnicity

**DOI:** 10.3390/jcdd12060199

**Published:** 2025-05-23

**Authors:** Narayanaswamy Venketasubramanian

**Affiliations:** Raffles Neuroscience Centre, Raffles Hospital, Singapore 188770, Singapore; drnvramani@gmail.com or ramani_nv@rafflesmedical.com; Tel.: +65-6311-1111

Stroke remains a leading cause of death and disability globally. However, despite recent and continuing research into the epidemiology and management of stroke, there remain many knowledge gaps that impact on patient care. Disparities in stroke mechanisms and burden even within a country, usually attributed to differences in stroke risk factors, may well be related to ethnicity. There is continuing interest in unravelling the poorly studied implications of non-acute brain MRI findings; understanding severe organ dysfunction; prognosticating after hyperacute interventions not only based on brain imaging but also the function of other organs, diagnosis and management of arrhythmias such as atrial fibrillation to reduce stroke and systemic embolism; diagnosing stroke mechanisms such as paradoxical embolism, which is more common among the young; and reducing stroke recurrence with its additive effects on mortality and disablement. These exciting and clinically relevant matters are explored in this issue of *JCDD*, yielding new information that helps fill some of the knowledge gaps.

There is ongoing research on how and why strokes occur, largely based on epidemiological studies [1]. Strokes have been attributed to the traditional vascular risk factors of ageing, hypertension, diabetes mellitus, hyperlipidaemia, and cigarette smoking. However, not all populations show the same degree of incidence and mechanism of stroke even after adjusting for differences in stroke risk factors, which raises the possible role of ethnicity [2], and remains understudied outside of North America and Western Europe. Among ethnic comparative studies, Venketasubramanian presents a single-centre study of stroke demographics, risk factors, subtypes, syndromes, and mechanisms and explores inter-ethnic differences between Chinese, Malays and Indians hospitalised for stroke in the multi-ethnic Asian country of Singapore. It is noteworthy that intracerebral haemorrhage (ICH) was associated with ethnicity, being lowest among the Indians even after adjusting for vascular risk factors. In a health database study by Alysha et al. comparing Vietnamese-born with Australian-born residents in South-Western Sydney, Australia with transient ischaemic attacks (TIAs) or stroke, Vietnamese participants had a significantly higher frequency of hypertension, dyslipidaemia, ICH and intracranial atherosclerosis, while Australians had a higher frequency of smoking and alcohol abuse. An intriguing Mendelian randomisation analysis by Zhang et al. among East Asians (Chinese, Japanese, Koreans) revealed that a genetic predisposition to chronic hepatitis B was associated with a lower risk of large artery atherosclerotic stroke, but not other stroke mechanisms.

Neuroimaging plays a key role in the diagnosis and guidance for hyperacute management of stroke [3]. A hyperdense artery (HDA) seen on a CT brain scan may indicate the presence of a thrombus and could guide emergent revascularization therapies such as immediate endovascular thrombectomy (EVT) [4]. However, the clinical impact of HDA and other neuroimaging parameters on stroke recovery and outcome is still uncertain [5]. Marginean et al. performed a single-centre retrospective cohort study of patients with acute ischemic stroke (AIS) caused by large vessel occlusion. They identified a total of 21 different textural parameters on the non-enhanced CT scan that could predict the outcome of a thrombectomy procedure, but they had variable sensitivity and specificity. Dvornikova et al. published the design of a prospective multi-centre observational study of patients with AIS that would investigate various CT parameters that could impact on 3-month outcomes—the results of this study are awaited. In another outcome study, in their single-centre retrospective cohort study of AIS patients with large vessel occlusion who underwent EVT, Li et al. found that left ventricular diastolic dysfunction on echocardiography performed within 6 months after stroke onset was associated with poor functional recovery at 3 months but not in-hospital mortality.

Cardioembolism causes approximately 20% of strokes, with atrial fibrillation (AF) being the most common mechanism [1]. Anticoagulation has been proven to reduce recurrent stroke risk among patients with AF [3], but it is often underutilised due to fears of bleeding risks—newer therapies are becoming available [6]. Still, AF detection in the acute stroke care setting can be challenging in the absence of appropriate cardiac monitoring devices [7]. Patel et al. review and provide insights and strategies for optimised care for the diagnosis and management of AF in AIS in the setting of reperfusion therapy (intravenous thrombolysis IVT and EVT). In Ton et al.’s multi-centre registry-based study in Vietnam on patients hospitalised for TIA or ischemic stroke, those with AF were older and had more severe strokes, increased mortality and poorer outcome; their utilisation of anticoagulation therapy was significantly increased at discharge compared to pre-hospitalisation, with direct oral anticoagulants (DOACs) preferred—this is in line with current clinical practice guidelines [3]. Percutaneous left-atrial appendage closure (LAAC) is an alternative to oral anticoagulation for preventing strokes in patients with AF [6]. Kayvanpour et al. performed a retrospective analysis in their single-centre study comparing different closure devices. They found a very high procedural success rate, with cactus anatomy being the most challenging. One device had a significantly higher puncture-site bleeding rate, while significant peri-device leaks were more frequent among devices with a simple-sealing mechanism compared to those with double-sealing; there were no significant differences between the devices for thromboembolic outcomes or mortality.

Paradoxical embolism via a patent foramen ovale (PFO) may be a cause of otherwise cryptogenic stroke, especially among the young, who may not have the traditional vascular risk factors [1]. Kwok et al. performed a retrospective nationwide US analysis of the use a modified version of the Risk of Paradoxical Embolism (RoPE) score, where data on age, hypertension, diabetes mellitus, smoking and history of stroke or TIAs were retained, but the imaging data were not included, to allow its possible use in resource-poor settings. However, it was only modestly predictive of PFO, limiting its wider clinical applicability; interestingly, the strongest predictor was deep venous thrombosis.

The clinical significance of enlarged perivascular spaces (PVSs) seen on neuroimaging is uncertain. While they may be associated with increasing age, hypertension, lacunar infarcts and cerebral microbleeds, the between-study heterogeneity has led to uncertainty as to their impact on the patient [8]. In their prospective cohort study of patients with a recent small subcortical infarct, Sozzi et al. found that basal ganglia (BG)-PVSs were associated with lacunes, white matter hyperintensities and microbleeds; white matter (WM)-PVS were associated with lacunes; and brainstem PVS showed no associations. The WM/BG-PVS ratio was associated with lacunes, and interestingly, the Pittsburgh Sleep Quality Index suggested that sleep quality might impact on waste removal processes in different parts of the brain.

Treatment options of acute stroke patients may be limited by severe renal dysfunction (SRD). This includes the conduct of acute neuroimaging studies such as contrast-enhanced brain imaging via CT scan; CT angiography to detect arterial occlusion; CT perfusion studies to determine the existence of salvageable ischaemic penumbra; and performing cerebral angiography and EVT due to the nephrotoxicity of CT-contrast; in addition, DOACs are contraindicated in severe SRD due to many being significantly cleared by the kidneys [3,4,6]. It is difficult to predict based on pure clinical grounds which patient with no history of renal disease may have SRD. Mori et al. found in their a single-centre registry-based study that SRD, based on the Cockcroft–Gault equation for calculating the creatinine clearance, was more frequent among females, and that SRD was associated with loop diuretics and anaemia in both sexes. This finding will help raise the clinical suspicion of possible SRD among patients with these factors present even before laboratory results are available, which may take several hours.

Future research could investigate the ethnicity and incidence of stroke in South America, sub-Saharan Africa and among indigenous populations. Multi-centre studies are required to study the parameters on non-enhanced CT scans or echocardiography that could predict the outcome of EVT. Better strategies for optimised care are needed in the diagnosis and management of AF in AIS in the setting of reperfusion therapy and the prescription of evidence-proven DOACs. Multi-centre, blinded, randomised, controlled head-to-head trials should be performed comparing LAAC devices among patients with AF.

There is strong and continued interest in conducting research in cardio and cerebrovascular disease. *JCDD* will continue to publish high-quality—submissions are always welcome.
